# Automatic Number Plate Detection and Recognition System for Small-Sized Number Plates of Category L-Vehicles for Remote Emission Sensing Applications

**DOI:** 10.3390/s25113499

**Published:** 2025-05-31

**Authors:** Hafiz Hashim Imtiaz, Paul Schaffer, Paul Hesse, Martin Kupper, Alexander Bergmann

**Affiliations:** Institute of Electrical Measurement and Sensor Systems, Graz University of Technology, 8010 Graz, Austria; paul.schaffer@tugraz.at (P.S.); paul.hesse@student.tugraz.at (P.H.); martin.kupper@tugraz.at (M.K.); alexander.bergmann@tugraz.at (A.B.)

**Keywords:** automatic number plate detection and recognition, motorcycle number plate detection, image processing, computer vision, convolutional neural networks, remote emission sensing

## Abstract

Road traffic emissions are still a significant contributor to air pollution, which causes adverse health effects. Remote emission sensing (RES) is a state-of-the-art technique that continuously monitors the emissions of thousands of vehicles in traffic. Automatic number plate recognition (ANPR) systems are an essential part of RES systems to identify the registered owners of high-emitting vehicles. Recognizing number plates on L-vehicles (two-wheelers) with a standard ANPR system is challenging due to differences in size and placement across various categories. No ANPR system is designed explicitly for Category L vehicles, especially mopeds. In this work, we present an automatic number plate detection and recognition system for Category L vehicles (L-ANPR) specially developed to recognize L-vehicle number plates of various sizes and colors from different categories and countries. The cost-effective and energy efficient L-ANPR system was implemented on roads during remote emission measurement campaigns in multiple European cities and tested with hundreds of vehicles. The L-ANPR system recognizes Category L vehicles by calculating the size of each passing vehicle using photoelectric sensors. It can then trigger the L-ANPR detection system, which begins detecting license plates and recognizing license plate numbers with the L-ANPR recognizing system. The L-ANPR system’s license plate detection model is trained using thousands of images of license plates from various types of Category L vehicles across different countries, and the overall detection accuracy with test images exceeded 90%. The L-ANPR system’s character recognition is designed to identify large characters on standard number plates as well as smaller characters in various colors on small, moped license plates, achieving a recognition accuracy surpassing 70%. The reasons for false recognitions are identified and the solutions are discussed in detail.

## 1. Introduction

Emissions from road traffic significantly contribute to air pollution, which poses a serious risk to public health [1]. Emissions per vehicle have been significantly reduced as vehicle manufacturers follow stricter limits and produce engines with advanced after-treatment methods to reduce vehicle exhaust emissions. Moreover, people are moving towards electric vehicles and reducing the use of internal combustion engine (ICE) vehicles. Even with these restrictions and the increasing use of electric cars, high-emitting ICE vehicles still contribute significantly to air pollution. About 90% of road pollution comes from 15% of high-emitting vehicles [2], and internal combustion engine (ICE) cars will remain on the roads for at least 30 more years [3]. Thus, continuous monitoring of on-road traffic is necessary to detect high emitters. Remote emission sensing (RES) is a state-of-the-art methodology to monitor on-road traffic and detect high emitters. With the RES systems, we can monitor thousands of on-road vehicles daily. Automatic number plate recognition (ANPR) systems are crucial in identifying high emitters within remote emission sensing (RES) systems. ANPR systems enable retrieving essential vehicle information, i.e., vehicle type, brand, model, manufacture year, fuel type, emission standard, and power, from vehicle registration databases. False recognitions and errors in ANPR systems lead to invalid emission measurements, as we cannot link the emissions to the corresponding vehicle. Recognizing number plates on Category L vehicles (two-wheelers) presents additional challenges because of variations in number plate size and placement across different categories. In some countries, the number plates on mopeds are tiny and come in various colors, making them difficult to read with ANPR systems designed for standard vehicles with conventional-sized plates. Therefore, there is need to develop a specialized ANPR system tailored to recognize L-vehicle number plates. This work focuses on creating a Category L vehicle automatic number plate recognition (L-ANPR) system to accurately identify number plates of differing sizes and colors from different categories and countries.

RES methodology has been used to detect high emitters since the end of the 20th century. RES techniques are of two types: extraction-based and absorption-based. In extraction-based RES systems, also called point sampling (PS), the sampling line is placed on the side of the road to extract the diluted exhaust of passing vehicles. In 1990, Hansen et al. used the PS technique to measure carbon dioxide (CO_2_) and black carbon (BC) in the plumes of passing on-road vehicles [4]. In comparison, advanced RES systems use absorption spectroscopic techniques to detect emissions from the exhaust plumes of passing vehicles. These systems consist of a laser transmitter and a receiver placed roadside. In 1989, Bishop et al. introduced the first operational horizontal RES system [5]. Since then, the RES technique has been used in various measurement campaigns to detect high emitters. In 2005, Janhäll et al. used an extraction-based RES system to measure pollutants in vehicle exhaust at four different sites in Gothenburg, Sweden [6]. In 2013, Hallquist et al. used the RES technique to detect emissions from buses [7]. In 2023, Ghaffarpasand et al. shared analysis of the emission data collected from commercial RES systems used in RES campaigns in five different urban UK environments [8]. From 2019 to 2023, during the CARES project, RES systems monitored hundreds of thousands of vehicles in Krakow, Milan, and Prague [9]. In 2024, Knoll et al. evaluated the black carbon tracker using a point sampling method and tested thousands of passing vehicles in multiple European cities [10], and Imtiaz et al. developed an imaging system for qualitative and quantitative analysis of vehicle exhaust plumes for RES applications [11].

Recording number plates to link emissions to the associated vehicles is crucial to RES measurements. In 1990, Stedman and Bishop analyzed RES and described the first commercial absorption-based RES system, FEAT (fuel efficiency automobile test) [12]. At that time, the FEAT system did not have a separate ANPR system. It had a video recording camera, and data on vehicle number plates were captured manually from the videos. ANPR systems were costly to include in RES systems at that time. The first ANPR was invented in 1976 in the United Kingdom [13]. Ahmad et al. compare different ANPR techniques and their advantages [14], and over time, numerous innovative strategies have been introduced to boost the effectiveness of ANPR systems significantly. Paruchuri further presents an overview of the application of artificial neural networks to ANPR [15]. In 2019, a portable ANPR system on Raspberry Pi was proposed by Fakhar et al. [16]. In 2020, Nayak et al. described the importance of advanced optical character recognition algorithms in the ANPR system [17]. In 2021, Salma et al. used convolutional neural networks to develop an ANPR system [18]. In 2024, Rafek et al. used the deep learning model YOLOv4 for number plate recognition in video streams [19]. Mahmud Al-Hasan et al. proposed an enhanced YOLO v8-based system for ANPR [20]. Also in 2024, Ruiyang Liu proposed an improved LKM-YOLOv10 vehicle license plate recognition detection system based on YOLOv10 [21], and Zunair et al. proposed a high-resolution dataset of twenty thousand images for road scene understanding in autonomous driving. In those studies, they benchmarked state-of-the-art object detectors and explored large image models as image annotators. The state-of-the-art object detectors include YOLOv6, YOLOv8, and transformer-based DETR and RTMDET. According to the studies, YOLOv6 and YOLO8 have the highest accuracy [22]. In 2025, A.S. Geetha reported that the YOLOv4 object detector achieved the best performance on the COCO dataset. This was accomplished by integrating advanced techniques for both regression (bounding box positioning) and classification (object class identification) within the Darknet framework. This development is considered a breakthrough in real-time object detection [23].

ANRP systems are a crucial part of the RES system. Many commercialized state-of-the-art ANPR systems are available today to detect and recognize vehicle license plates. To the best of our knowledge, there is no ANPR system specially designed for Category L vehicles, and there is very little information on the usage of ANPR systems on small, moped license plates, the associated problems, and solutions.

- In this work, generic state-of-the-art license plate detection and recognition algorithms are tested with different types of Category L vehicles, and a performance comparison is presented.
- A cost-effective and energy efficient Category L vehicle automatic number plate recognition (L-ANPR) system is developed to detect and recognize license plates on Category L vehicles.
- The L-ANPR system recognizes L-vehicles by calculating the size of passing vehicles using photoelectric sensors (light barriers), as L-vehicles are smaller than cars. After identifying an L-vehicle, the light barriers trigger the L-ANPR detection system and emission measurement devices to start monitoring. This technique significantly reduces energy consumption, data usage, data storage, and computational costs.
- The L-ANPR system’s convolutional neural network-based automatic detection model is trained with thousands of images of license plates of different types of Category L vehicles from different countries. The training data are collected from public online datasets, remote emission sensing device validation campaigns, and remote emission measurement campaigns performed in different European countries. The datasets were appropriately annotated for training, and the trained weights were generated. The advantage of the automatic detection model is that this single model is capable of detecting tiny plates of different colors on mopeds as well as normal-sized car and bike plates.
- The L-ANPR character recognition system is designed to recognize characters from license plates of different types of Category L vehicles. Plate conditioning, background color-based thresholding, and dilating techniques are applied to increase the visibility of small characters from the background and improve the performance of the optical character recognizer for small plates. The advantage of a character recognition algorithm is that it can quickly recognize large characters from standard number plates as well as small characters of different colors from the tiny plates on mopeds from different countries. Additionally, the L-ANPR system can capture 90–120 frames per second, enabling recognition of as many characters as necessary by reviewing all frames during post-processing for any false recognition that occurred in real-time.
- Emission measurement campaigns have been conducted under the European Union-supported LENS project [24]. The L-ANPR system was implemented on roads and with remote emission sensing devices during validation campaigns in Austria and emission measurement campaigns in Belgium. It was tested with hundreds of Category L vehicles from different countries, and its performance was evaluated. Another advantage of implementing L-ANPR in the emission measurement campaigns is that a large amount of data on different types of L-vehicles was collected, which was then used for further training and improving the performance of the L-ANPR system.
- After evaluating the performance of the L-ANPR system, the reasons for false recognitions are highlighted, and solutions are proposed to improve the performance of ANPR systems in detecting the tiny plates of L-vehicles.

The design of the L-ANPR system and its detection and recognition algorithms are described in detail in the following section.

## 2. Materials and Methods

### 2.1. Roadside Emission Measurement Setup

**Figure 1:**
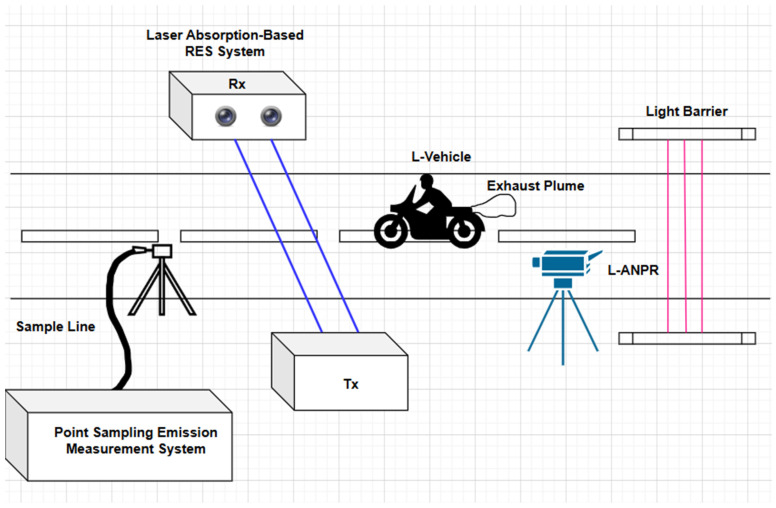
Remote emission sensing road setup schematic: Light barriers are placed along the roadside to calculate the size of vehicles, identify L-vehicles, and trigger the systems to start monitoring. Point sampling-based and laser absorption spectroscopic-based RES systems are placed along the roadside to measure emissions. The L-vehicle automatic number plate detection and recognition system is also placed along the roadside to recognize high emitters.

A roadside emissions measurement setup may consist of a RES setup based on laser absorption spectroscopy, or a point sampling setup. Mandatory components include light barriers and an ANPR system for L-vehicles, as shown in Figure 1. A point sampling system consists of a sample line placed alongside the road to extract the exhaust of the passing vehicle to measure emissions. In comparison, a laser-based RES system consists of a laser transmitter and receiver placed roadside. The laser passes through the vehicle’s exhaust and reaches the transmitter after being absorbed in the exhaust plume. When a vehicle passes the light barriers, the RES and ANPR systems are triggered to start measurements.

### 2.2. L-Vehicle Automatic Number Plate Detection and Recognition (L-ANPR) System

**Figure 2:**
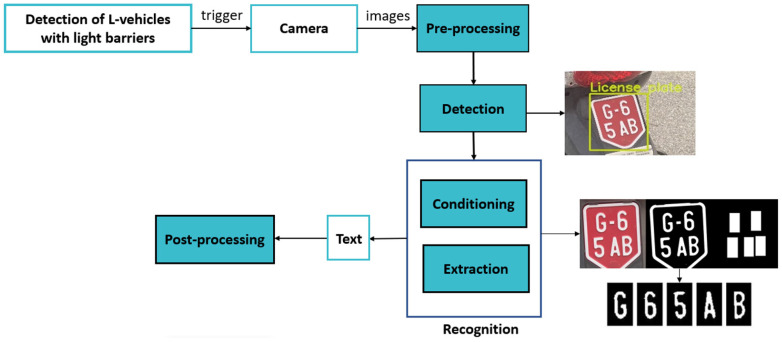
L-vehicle automatic number plate detection and recognition algorithm data flow.

The L-ANPR system takes images directly from the camera and outputs text in the form of a string of characters. The main hardware of the L-ANPR system is the camera, which captures and feeds images to the processing unit. The pre-processing step serves to remove adverse weather and low-light effects from the test images, as described in Section 2.6.1. The conversion of images to text is achieved by image processing and deep learning techniques. The conversion consists of two main algorithms, Detection and Recognition, which are implemented in the processing unit, as shown in Figure 2. The L-ANPR hardware components and detection and recognition algorithms are discussed in detail in the following sections.

### 2.3. Hardware for L-ANPR

Cameras—The L-ANPR system consists of two cameras. The first camera is a 12.3 MP Raspberry Pi HQ model with a Sony IMX-477R sensor. The camera is produced by the Raspberry Pi Foundation, based in Cambridge, England. The camera sensor is produced by Sony Semiconductor Solutions Corporation, located in Atsugi, Japan. This sensor offers a maximum resolution of 4056 pixels horizontally by 3040 pixels vertically, with a single pixel size of 1.5 μm by 1.5 μm. It can output in 8-, 10-, or 12-bit RAW formats. The camera has an adjustable focus and allows for attaching lenses with varying focal lengths, compatible with C/CS mounts. It operates within the visible light wavelength range of 350 to 750 nm. Additionally, the device is equipped with a rolling shutter. It can read up to 840 million pixels per second, capturing between 15 to 240 frames per second, depending on the resolution setting. The second camera is a 1.6 MP Raspberry Pi Global Shutter Camera with a Sony IMX 296 sensor. The sensor size is 6.3 mm diagonal with a single pixel size of 3.45 μm by 3.45 μm. The camera is also compatible with C/CS mounts, using lenses of different focal lengths. It can output in 10-bit RAW formats. The cameras have a global shutter, which captures fast-moving objects without distortion. The cameras use lenses with 16 mm and 6 mm focal lengths. There is no need for an external power supply to power the cameras. Instead, the cameras take power directly from the Raspberry Pi, making them more energy efficient.

Light Barriers—Photoelectric sensors, transmitters, and receivers from RS Pro are used as light barriers to calculate the speed, acceleration, and size of passing vehicles and to trigger the other systems to start monitoring after recognizing L-vehicles. The sensors use red light (630 nm) for detection. The sensing distance range is 10 m. The sensors are manufactured by RS Group, which includes the RS PRO brand and has its headquarters in Corby, Northamptonshire, UK.

Control Unit—The L-ANPR system uses Raspberry Pi 4B as control unit for the cameras. The Raspberry Pi 4B is manufactured by the Raspberry Pi Foundation, based in Cambridge, England. It has a 64-bit Quad Core ARM v8 Cortex-A72 processor and 8 GB RAM. It has Gigabit Ethernet and onboard wireless networking. It provides support for dual displays with resolutions up to 4 K through two micro-HDMI ports and hardware video decoding capabilities of up to 4 Kp60. It generally requires about 2.5 W to 5 W during standard operation.

GPU—An external GeForce GPU with plate number RTX-3080 is used to efficiently implement convolutional neural network-based detection algorithms. The GPU has 8960 NVIDIA CUDA Cores with a Boost Clock of 1.71 GHz and a Base Clock of 1.26 GHz, which offers the advantage of fast parallel processing. It contains 12 GB GDDR6X-RAM with a memory interface width of 384-bit. The GPU GeForce RTX-3080 is manufactured by NVIDIA, which is located in Santa Clara, California, United States.

**Figure 3:**
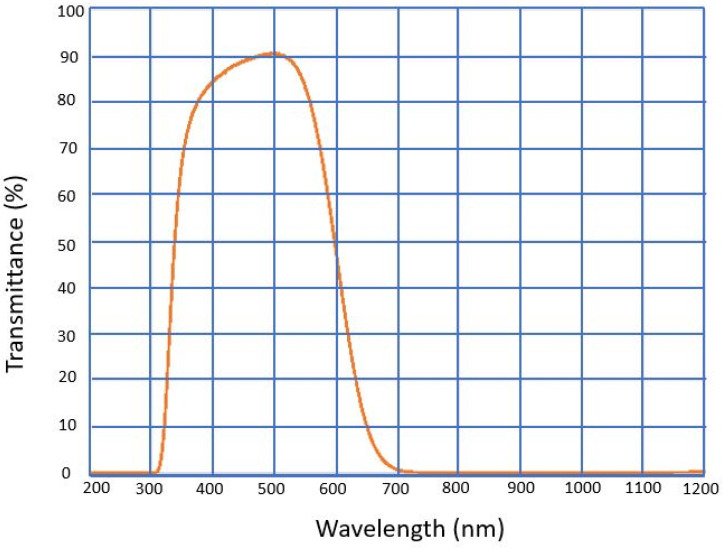
Transmission characteristics of the IR filter used in Raspberry Pi HQ cameras.

IR Filter—The Raspberry Pi HQ and Global Shutter Cameras have a built-in Hoya CM500 infrared filter (IR). The transmission characteristics of the IR filter are represented in Figure 3 [25]. The filter reduces the sensitivity of the cameras to the IR light. The filter can be removed to provide night vision if the location is illuminated with IR light. It is necessary to remove the IR filter to improve the L-ANPR’s nighttime performance. The procedure for removing the IR filter is described in [26].

### 2.4. Detection of L-Vehicles with Light Barriers of L-ANPR System

**Figure 4:**
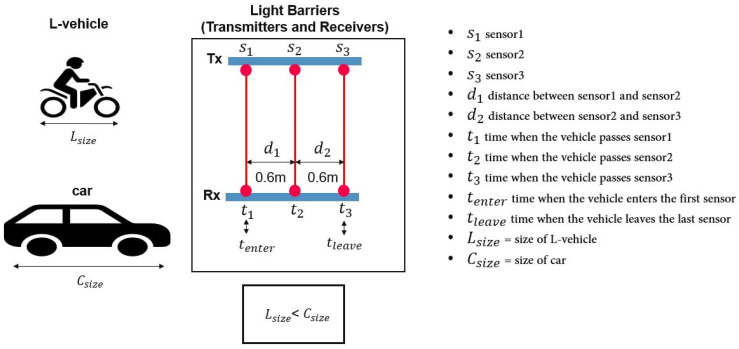
Schematic of L-ANPR system light barriers.

L-ANPR system includes light barriers (light transmitters and receivers) to recognize category L-vehicles by calculating the size of passing vehicles. The schematic of the light barriers is shown in Figure 4. L-vehicles are smaller than cars and trucks, so they can be identified based on size. After identifying an L-vehicle, the light barriers trigger the L-ANPR detection system to start capturing frames for detection. This technique saves a significant amount of energy, data, and computational costs. Moreover, the speed and acceleration of the passing vehicle can be calculated with the light barriers.

#### Calculation of Size of Passing Vehicles

An array of three pairs of photoelectric sensors (s_1_, s_2_, and s_3_) are used to calculate the size of passing vehicles. The distance between the sensors s_1_ and s_2_ is d_1_, and the distance between the sensors s_2_ and s_3_ is d_2_. Times t_1_, t_2_, and t_3_ are when the vehicle passes the sensors s_1_, s_2_, and s_3_, respectively. The velocity of the passing vehicle V_pass_ is calculated first using equation 1 to calculate the size:

V_pass_ = (d_1_ + d_2_)/(t_3_ − t_1_) (1)

The size (S) of the vehicle is calculated using the time (t_enter_) when the car first hits the first sensor s_1_, the time (t_leave_) when the car leaves the last sensor s_3_, and the velocity of the car, using Equation (2):

S *=* V_pass_ × (t_leave_ − t_enter_) (2)

### 2.5. L-ANPR License Plate Detection

**Figure 5:**
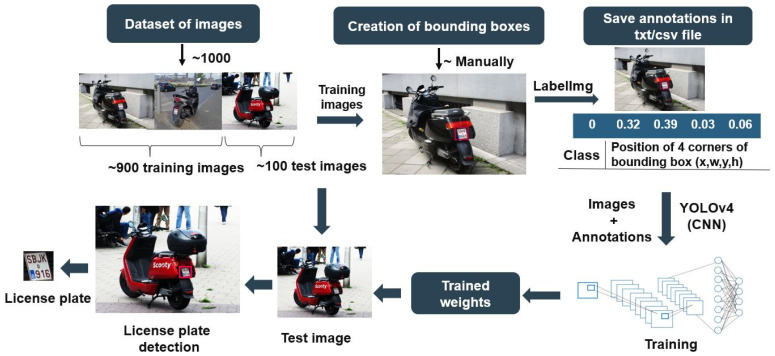
L-vehicle automatic number plate detection algorithm flow diagram.

License plate detection is the first and most essential stage of any ANPR system. The detection algorithm of the L-ANPR is based on a convolutional neural network and consists of different steps, as shown in the flow diagram in Figure 5. The steps are explained below in detail.

#### 2.5.1. Dataset of License Plate Images

**Figure 6:**
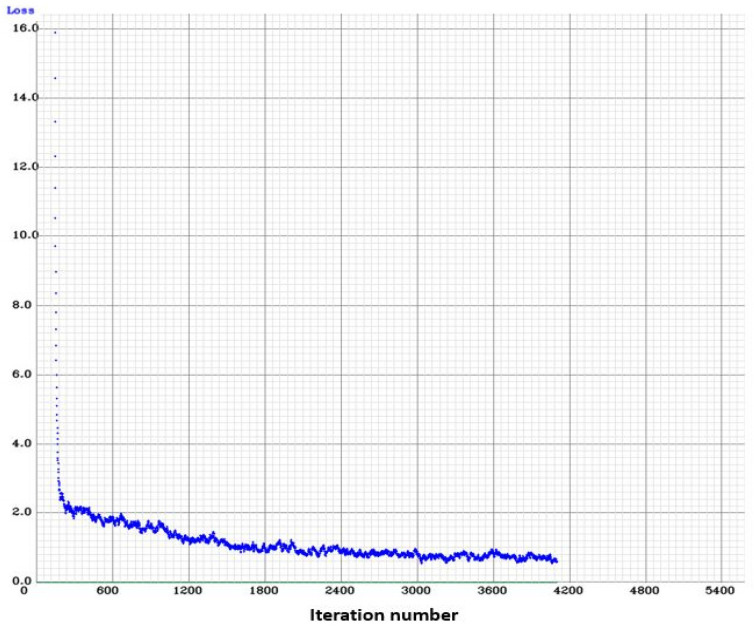
Loss plot during L-ANPR detection model training.

The algorithm for the automated detection of license plates from images must be trained. The algorithm learns from training data, and the quality of training data is crucial for good detection rates. Publicly available L-vehicle datasets consist mainly of larger bikes with large number plates. The challenging, tiny number plates on mopeds are not included in most publicly available datasets. We captured images of small, moped number plates in Austria and Belgium and included them in our training data. Our training dataset comprises around 1000 images of Category-L vehicle license plates. The training images were taken from publicly available datasets [27] and captured at validation and emission measurement campaigns. The dataset is divided into a training images set and a testing images set. The training images set contains about 900 images, while the testing images set contains around 100 images. All data is treated in accordance with General Data Protection Regulations (GDPR). The loss plot during training is shown in Figure 6, and demonstrates how our model performed after each training iteration. The loss plot shows the loss value or the value of our error function for each training iteration. The configuration for training an L-ANPR detection model is shown in Table 1. The precision, recall, and F1-score of the L-ANPR detection model is shown in Table 2.

#### 2.5.2. Creation of Bounding Boxes

Training images and annotations are needed to train the detection algorithm. The annotations are the coordinates of the bounding box or label around the object we want to detect in an image. Labeling of the training images is performed and annotations are created using LabelImg 1.8.6 software [28].

#### 2.5.3. Object Detection Model—YOLOv4

YOLOv4 is a state-of-the-art convolutional neural network (CNN)-based real-time object detector designed to balance speed and accuracy [29]. It uses CSPDarknet53 as a backbone, which contains 29 convolutional layers 3 × 3 with 27.6 M parameters [30]. The training images and created annotations are given to the YOLOv4 model, and trained weights are generated. In CNN, the weights are the kernels used to perform the convolution operation in the convolutional layers of the neural network. The weights’ values are randomly initialized and then updated and optimized after learning from the training data. The trained weights are then applied to the test images to detect license plates and the corresponding annotations.

YOLOv4 is designed to run on a single GPU and uses CSP (Cross-Stage Partial) NetDarknet53 as a backbone to extract features. The CSP strategy reduces the number of feature maps that go through the network and uses less RAM, which results in a 20% reduction in computation. YOLOv4 uses a modified PAN (Path Aggregation Network) technique and passes features from lower levels to back up through the network, which increases the performance. The modified PAN technique is more efficient for single GPU training. YOLOv4 is one of the best choices for real-time and accurate object detection using a single GPU and low-resolution images (we used a resolution of 640 × 480 pixels) with less complexity compared to other state-of-the-art models.

#### 2.5.4. Detection with Test Images

The trained optimized weights are applied to the test images to detect license plates and calculate the corresponding annotations. The annotations are then saved in a text file for future use. The annotations are used to crop the image to the region of interest. The images from the testing images set are used for detection testing, and random images are also tested using the detection algorithm.

### 2.6. L-ANPR Character Recognition

**Figure 7:**
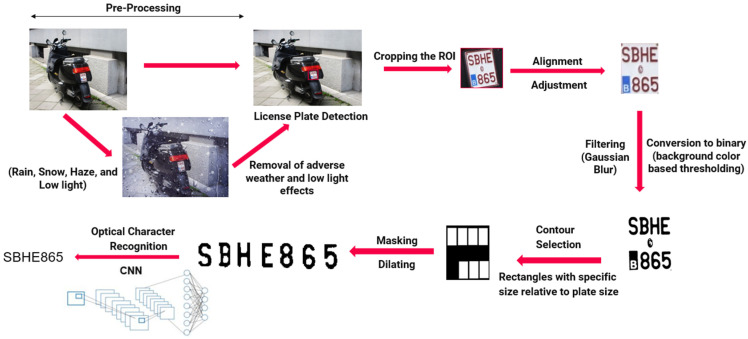
L-vehicle automatic number plate recognition algorithm flow diagram.

After the license plate is detected from images of vehicles on the road, the correct recognition of all characters is necessary to identify the number of the license plate. The license plates of mopeds in some countries are very small, and the characters are even smaller. Our character recognition algorithm is designed for all types of L-vehicle license plates. The recognition algorithm consists of two parts, Conditioning and Extraction, which are explained below. The flow diagram containing the necessary steps for character recognition is shown in Figure 7.

#### 2.6.1. Pre-Processing: Removing Adverse Weather and Low Light Effects

The license plates of certain Category L vehicles are quite small. Weather conditions such as snow, rain, haze, and low light hinder the visibility of these small plates, making them difficult to read and recognize. This also decreases the effectiveness of number plate detection and character recognition algorithms, as shown in Figure 7. The pre-processing step serves to remove adverse weather and low-light effects from the test images using state-of-the-art algorithms. In 2022, Chen et al. proposed a novel approach for removing the effects of bad weather using the unified model [31]. In 2023, Yang et al. proposed a language-driven restoration (LDR) framework for removing the effects of adverse weather conditions [32]. In 2025, Liu et al. proposed a variational nighttime dehazing framework, VNDHR, using hybrid regularization [33].

#### 2.6.2. Plate Conditioning

No matter how the actual reading from the plate is done, computer vision techniques are essential for providing good input to the algorithms reading from the image. Plate conditioning consists of several steps, which are explained in detail below. The steps include cropping the region of interest (ROI), alignment, removing noise, changing the color scale, and thresholding. This step is also often referred to as character segmentation.

Cropping to the Region of Interest—The second step is to extract the region of interest (ROI) obtained in the plate detection from the source image and resize the ROI. This step is necessary so that the resulting image of just the number plate always has the same dimensions, no matter how significant the ROI in the source image really is. The size was chosen to keep the image relatively small, yet still provide extensive-enough pixel density for good performance of the optical character recognition system.

Alignment and Adjustment—Figure 1 shows the position of the L-ANPR. Because the L-ANPR system is placed roadside, it is not possible to capture images of passing vehicles at a 0° angle; the license plates of passing L-vehicles in the captured images are tilted to some angle. As the license plates of mopeds are very small, the resolution of the license after cropping the region of interest is very low. Very small, tilted characters with very low resolution would be complex to recognize by an optical character recognition algorithm. To overcome this potential issue in a later stage, the license plates are angled to 0°. After alignment, the plates are adjusted to the region of interest by removing extra pixels around the corners.

**Figure 8:**
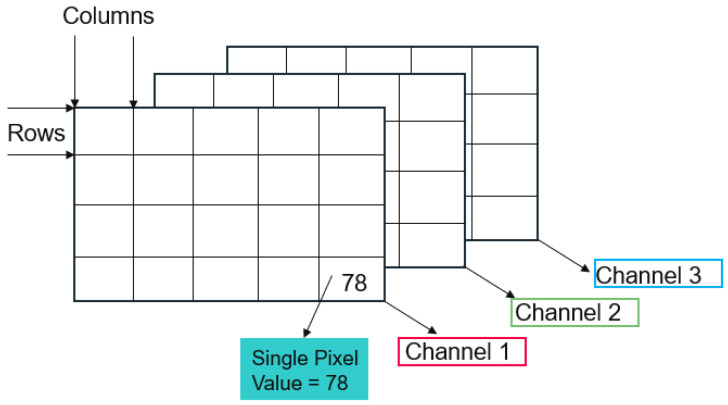
Schematic of composition of an RGB image.

Color Scales—Understanding color scales requires knowledge of images from a data perspective, as explained in [34]. A simple black-and-white image can be interpreted as a matrix, where the number of columns represents the width and the number of rows is the height of the image. Each matrix entry (pixel) holds a value corresponding to its intensity. The most common format, therefore, is 8-bit numbers, so each pixel has a value between 0 (black) and 255 (white). This range in values makes it possible to have binary images with just 0 and 255 as intensities and grayscale images with varying degrees of gray corresponding to the intensity value. One can also introduce color and, therefore, use not one, but multiple matrices to display an image as a superposition. Matrices combined into one picture are called bands or channels. Conventional color images are stored in the RGB format, where each main color (red, green, blue) has its own band with intensities ranging from 0 to 255. Figure 8 shows the general composition of an image. It is possible to use as many bands as needed, for example, a fourth channel just for infrared light.

**Figure 9:**
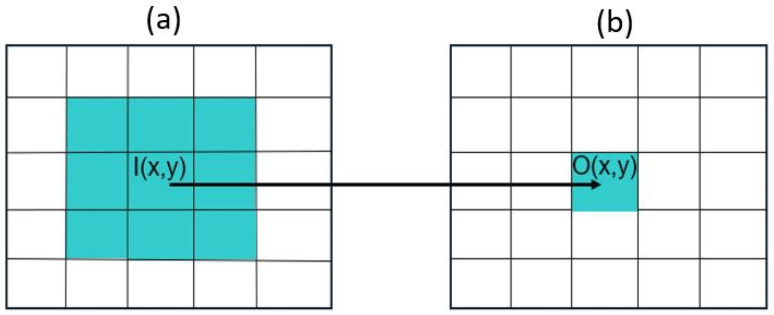
(**a**) The green area in the image is defined by the kernel (I), which contains 9 pixels and is represented by a 3 × 3 matrix. The filtering is applied to all pixels, and (**b**) the new pixel value (O) is calculated, which is then assigned to all 9 pixels.

Image Filtering and Gaussian Blur—Filtering an image involves manipulating its pixels in a certain way, with the goal of smoothing, noise reduction, and overall improved visual quality [35]. In Figure 9, a generic example of a filter is shown. The underlying concept is that the new value of each pixel depends not only on its original value but also on the pixel intensity values of the surrounding area. The size of this area is defined by the so-called kernel, which can be interpreted as a matrix. In Figure 9, this kernel is represented as a gray 3 × 3 square. The filtering itself is then applied to all pixels in the kernel (I), and the value of the new pixel (O) is calculated.

The most basic filter methods are the median filter, where the new pixel is the median of all pixels in the kernel, and the mean filter, where the new pixel is the mean of all the other pixels contained in the kernel. Of course, it is possible to vary the kernel size to tweak performance for the specific use case. Also, kernel entries can be chosen to weigh each pixel differently or according to distribution functions. Gaussian blur, which is used for the ANPR, weighs the pixels by incorporating the Gaussian Distribution Function.

Gaussian blur is a type of low-pass filter applied mathematically to the image to blur it. With Gaussian blur, the image is softened, uneven pixels in the image are smoothed by cutting out extreme outliers, and text becomes more apparent. The convolution of the image with the Gaussian function is applied to implement Gaussian blur. In the image, there is a lot of variation, which causes the pixels to have a high standard deviation. When Gaussian blur is applied to a group of pixels, a normal distribution of those pixel values is created, and the pixels are given new values equal to the weighted average of the surrounding pixels.

Thresholding—Thresholding is an image processing method that is used to binarize an image. If the value of a particular pixel is more significant than a previously defined threshold, its value changes to white (255); otherwise, it changes to black (0). Of course, this simple principle can be heavily improved. Using a kernel allows for calculating a threshold value for each pixel based on its surrounding pixels instead of relying on a single global threshold. This is essential for good results, as, most times, the illumination of an image is not uniform. Different methods exist to compute such adaptive or local thresholds, such as Otsu’s method [36], which was used for the L- ANPR.

Contour Detection—Contours play an essential role in the detection of objects located within images. In the case of ANPR, we search for rectangular contours because the number plates are rectangular. There are many definitions and approaches to finding contours [37]. One of the most common pixel-based approaches focuses on changes in pixel intensity: if the difference in intensity between two neighboring pixels exceeds a certain threshold, it is a possible contour. Of course, this approach leads to many single points. By interpolating these points with straight lines, contours reveal transitions between different objects in the image.

Masking and Dilating—Masking is performed to extract characters and remove background from the characters. Masking is applied using basic bitwise operations to the images and contours.

Dilating is the process of enhancing the brighter regions in the images. For dilation, the image is convolved with a square- or circle-shaped kernel and an anchor point in the center. The kernel overlaps the input image, and the maximum value is computed due to the kernel overlapping. The image pixel in the anchor point position is replaced with the calculated maximum value [38]. As a result, the area of characters dilates, becoming more visible and easier to recognize.

#### 2.6.3. Character Extraction and Recognition

Optical Character Recognition (OCR) describes methods to read text from images, such as extracting letters and numbers from a picture or video containing some text. There is a wide range of variants, from extracting machine-written fonts to even reading handwritten notes.

There are, of course, several different approaches for performing OCR, but they can be broken down into two categories [39]. The first is the technique of template matching, where the goal is to match predefined fonts to areas in the image containing the text. This approach yields high accuracy for machine-written text in an already-known font, but performance decreases significantly if the font is unknown. The second approach is called feature extraction, where features of characters are recognized, for example, horizontal lines in some characters or circle-like enclosures in letters. A model is then trained to correlate combinations of these features to the right character. This process benefits largely from the use of neural networks. The main downside to this approach is the high amount of training data necessary to develop the model. However, once trained, it provides high accuracy, even for fonts or handwritten notes that have never been seen before if trained on handwritten characters. Both methods can be combined, and machine learning enables intelligent OCR, where the software looks at single characters and attempts to read words and complete sentences to improve accuracy even further by applying grammar rules and semantic patterns [40]. The latter is the approach we followed for this work.

Considering the necessary effort for a well-performing OCR, it is evident that developing an OCR capable of achieving high accuracy was beyond the scope of this project. However, state-of-the-art OCR is available as open-source software, which allows one to focus on the pre-processing steps, then take advantage of a well-trained model later. The selected software is Tesseract OCR (version 5.3) [41,42], which fulfills all the requirements. Hewlett–Packard developed it in the 1990s and made it available as an open source in 2005. From 2006 until 2018, Google further developed it. Other state-of-the-art OCRs are easyOCR and keras-OCR, but Tesseract seemed the best fit.

### 2.7. Devices Validation Campaign L-ANPR Setup

**Figure 10:**
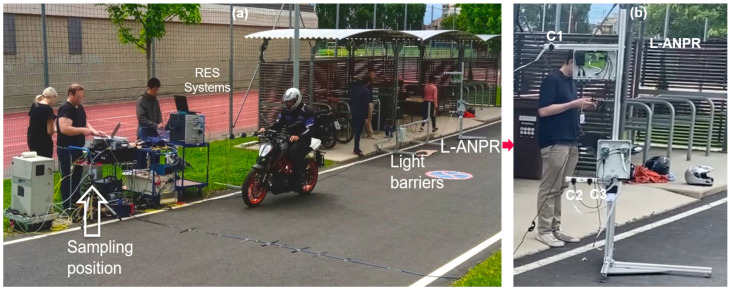
(**a**) Road setup of validation campaign at Graz University of Technology Inffeldgasse campus—The setup includes light barriers, point sampling-based remote emission sensing devices, and L-vehicle automatic number plate detection and recognition system. (**b**) A closer view of L-vehicle automatic number plate detection and recognition system with three cameras C1, C2, and C3 at different positions.

The RES, light barriers, and L-ANPR systems were set up at Graz University of Technology, Inffeldgasse Campus. The validation campaign was performed, and the systems were tested for different Category L vehicles. The complete validation setup with a passing bike is shown in Figure 10a. A closer look at the L-ANPR system is shown in Figure 10b. Three camera systems were used for validation. Cameras C1 and C2 are the Raspberry Pi HQ cameras, while C3 is the Raspberry Pi GS Camera. The cameras were tested at different heights with lenses of 6 mm, 16 mm, and 35 mm focal lengths. Various tests were performed with different types of Category L vehicles, and the optimal parameters for the L-ANPR system were identified.

### 2.8. Emission Measurement Camoaign L-ANPR Setup

**Figure 11:**
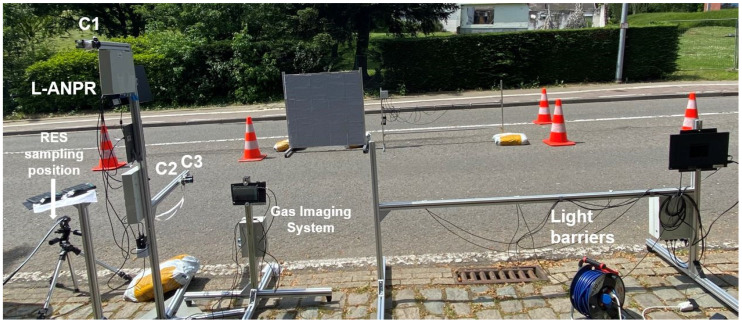
Road setup of L-vehicle emission measurement campaign at Donkerstraat, Leuven—The setup includes light barriers, point sampling-based remote emission sensing devices, and L-vehicle automatic number plate detection and recognition system.

A one-week emission measurement campaign was performed within the scope of the L-vehicles Emissions and Noise mitigation Solutions (LENS) project under the European Union’s Horizon Europe research and innovation program in Leuven, Belgium [43]. Validation and emission measurement campaigns were scheduled in the summer season, and sunny days were selected based on the assumption they were ideal for capturing a large number of Category L vehicles. The RES, light barriers, and L-ANPR systems were set up at Donkerstraat, Leuven, and hundreds of different Category L vehicles were tested with the systems. Measurements were taken over three days, with an average of 4 h of testing each day at this location. Measurements were also carried out over one day at the city center of Tiensestraat, Leuven. Measurements were taken when there was a high probability of passing L-vehicles. The systems setup is shown in Figure 11.

## 3. Results and Discussions

### 3.1. Performance Comparison of State-of-the-Art License Plate Detection and Recognition Algorithms on Category L Vehicles

**Figure 12:**
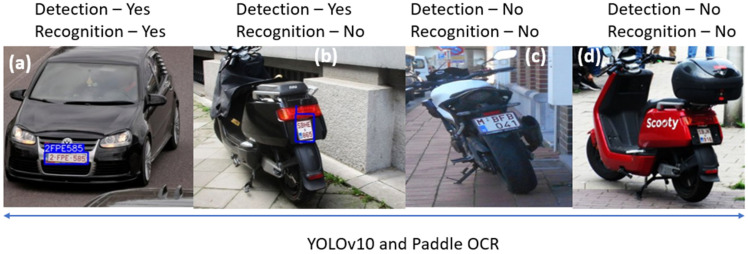
Testing an advanced automatic license plate detection and recognition algorithm based on YOLOv10 and Paddle OCR on different vehicles in Belgium: (**a**) Car, (**b**) Moped, (**c**) Bike, and (**d**) Moped.

**Figure 13:**
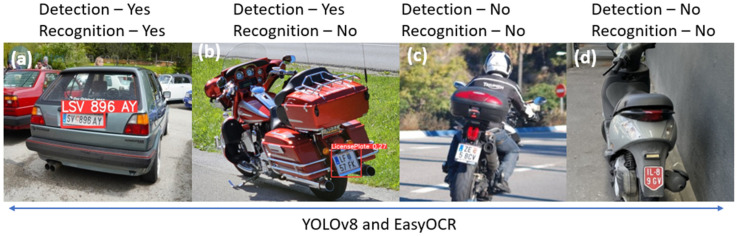
Testing an advanced automatic license plate detection and recognition algorithm based on YOLOv8 and Easy OCR on different vehicles in Austria—(**a**) Car, (**b**) Bike, (**c**) Bike, and (**d**) Moped.

The advanced license plate detection and recognition algorithms based on YOLOv8 [44], YOLOv10 [45], and Paddler OCR [46] are tested with the license plates of cars and Category L vehicles. Images of license plates were collected from online public datasets [47] and also from device validation and emission measurement campaigns. The algorithms performed well with car license plates, but they did not detect or recognize well the license plates on L-vehicles. The results are shown in Figure 12 and Figure 13. Some older ANPR systems that accurately recognize car license plates were also tested, but they failed to detect and recognize small moped plates effectively. Therefore, using generic ANPR systems on Category L vehicles is not optimal.

### 3.2. L-ANPR License Plate Detection

The L-ANPR license plate detection system was developed and tested with around 300 images of different L-vehicles. The testing images were collected from online datasets, a validation campaign performed in Graz, Austria, and an emission measurement campaign performed in Leuven, Belgium. The validation and emission measurement campaigns were scheduled during daytime on sunny days, because it was expected that these times would be ideal for capturing a large number of Category L vehicles. The overall detection accuracy of license plates on L-vehicles was around 90%, as shown in Table 3.

#### 3.2.1. Testing with Images from Online Datasets

**Figure 14:**
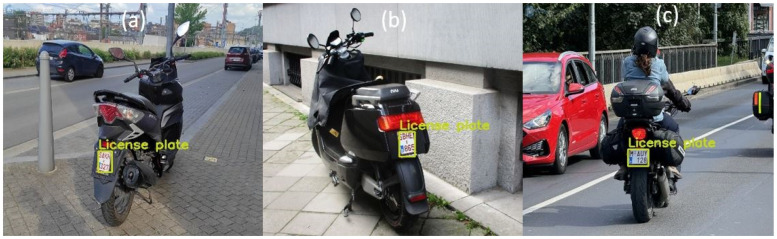
L-ANPR detection system applied to images from online datasets—(**a**) Moped, (**b**) Moped, and (**c**) Heavy bike.

The L-ANPR detection system was tested with various L-vehicle images of different types collected from public online datasets [48]. Figure 14a,b show the detection of moped license plates using the L-ANPR detection system, and Figure 14c shows the detection of the license plate on a large motorcycle.

#### 3.2.2. Testing with Images from Validation Campaign in Austria

**Figure 15:**
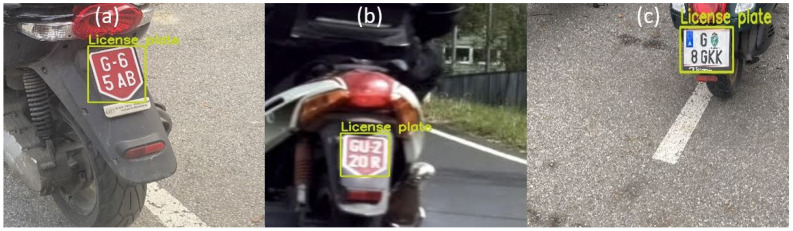
L-ANPR detection system applied to images from a validation campaign in Austria: (**a**) Moped, (**b**) Moped, and (**c**) Heavy Scooter.

A validation campaign was performed at Graz University of Technology, Inffeldgasse Campus. The L-ANPR detection system was tested with different types of L-vehicles around the campus. Figure 15a,b show the detection of the license plate of mopeds using the L-ANPR detection system, and Figure 15c shows the detection of the license plate on a large scooter.

#### 3.2.3. Testing with Images from Emission Measurement Campaign in Belgium

**Figure 16:**
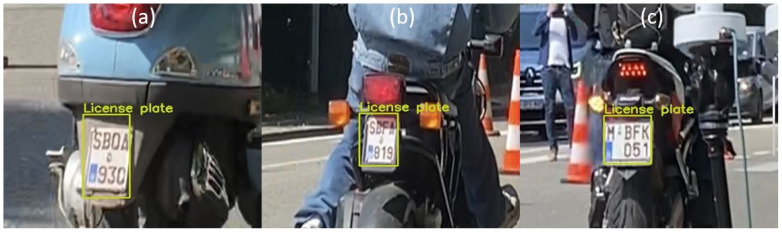
L-ANPR detection system applied to images from the emission measurement campaign in Belgium: (**a**) Moped, (**b**) Moped, and (**c**) Heavy bike.

An emission measurement campaign under the LENS project was performed in Donkerstraat, Leuven, Belgium. Hundreds of L-vehicles of different types were monitored during the campaign. Figure 16a,b show the detection of license plates on mopeds using the L-ANPR detection system, and Figure 16c shows the detection of the license plate on a large motorcycle.

### 3.3. L-ANPR License Plate Character Recognition

The L-ANPR license plate character recognition system was and tested with detected license plates on different types of L-vehicles received from the L-ANPR license plate detection system. The images of license plates were obtained from online datasets and captured during a validation campaign conducted in Graz, Austria, as well as an emission measurement campaign in Leuven, Belgium. The overall recognition accuracy for L-vehicle license plates was around 70%, as shown in Table 4. The recognition rate is lower, especially for images of license plates captured during the validation and emission measurement campaigns, due to lower picture resolution and the effects of shadow and sunlight. The reasons for false recognition and the possible solutions are described in Section 3.4.

#### 3.3.1. Testing with Images from Online Datasets

**Figure 17:**
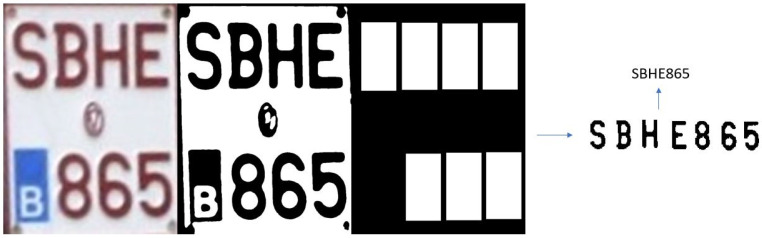
L-ANPR recognition system applied to a small moped license plate from an online dataset.

**Figure 18:**
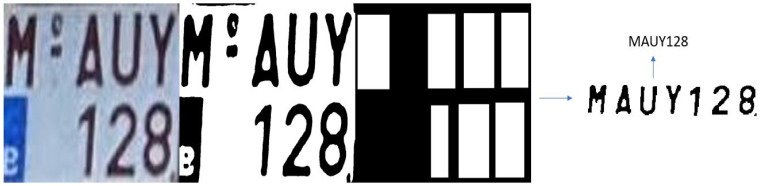
L-ANPR recognition system applied to the license plate of a heavy bike from an online dataset.

The L-ANPR recognition system was tested with images of license plates on different types of L-vehicles received from the L-ANPR detection system, and applied to images from online public datasets. The online images of license plates have excellent visibility and resolution, making recognizing characters easy. Figure 17 shows the recognition of characters on the license plate of a moped, while Figure 18 shows the recognition of characters on the license plate of a large motorcycle.

#### 3.3.2. Testing with Images from Validation Campaign in Austria

**Figure 19:**
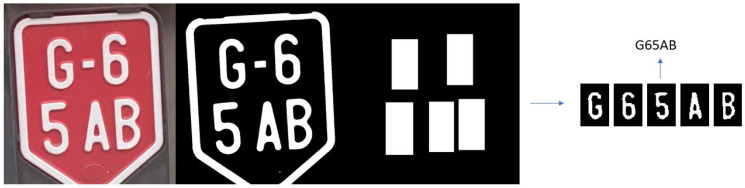
L-ANPR recognition system applied to a small moped license plate during the validation campaign in Austria.

**Figure 20:**
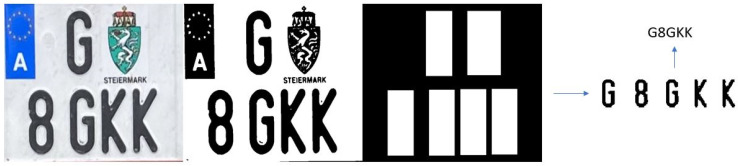
L-ANPR recognition system applied to the license plate of a heavy scooter during the validation campaign in Austria.

The idea of the validation campaign was to validate the remote emission sensing systems and L-ANPR system, and also to collect images of license plates on different L-vehicles around the Graz University of Technology, Infeldgasse campus for training and testing the algorithms and improve the performance of the L-ANPR system. The images of license plates were taken with different cameras, resolutions, and angles. Figure 19 illustrates character recognition on a moped license plate, while Figure 20 illustrates character recognition on a large motorcycle’s license plate.

#### 3.3.3. Testing with Images from Emission Measurement Campaign in Belgium

**Figure 21:**
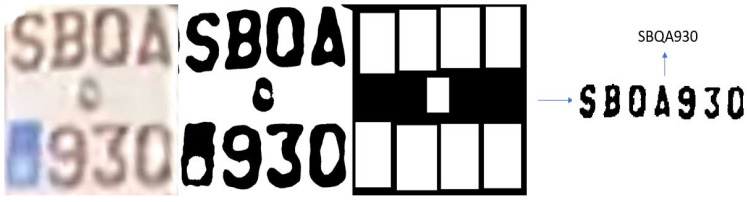
L-ANPR recognition system applied to a small moped license plate during the emission measurement campaign in Belgium.

**Figure 22:**
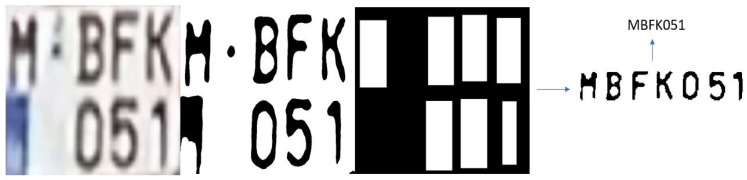
L-ANPR recognition system applied to the license plate of a heavy bike during the emission measurement campaign in Belgium.

The L-ANPR recognition system is tested with images of L-vehicle license plates captured during the emission measurement campaign in Leuven, Belgium. The photos were captured using Raspberry Pi HQ and Raspberry Pi GS models with a resolution of 640 × 480 pixels. The lower resolution was selected, taking 90–120 frames per second to capture the license plates of fast-moving L-vehicles. Figure 21 illustrates character recognition on a moped license plate, while Figure 22 illustrates character recognition on a large motorcycle’s license plate.

### 3.4. Limitations and Solutions

#### 3.4.1. Low Resolution License Plates

**Figure 23:**
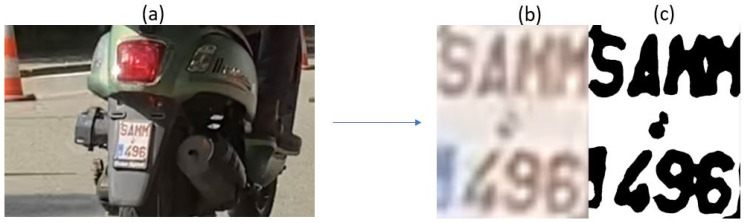
False recognition due to low resolution—(**a**) Image of passing vehicle, (**b**) Image of license plate after detection and cropping to region of interest, (**c**) Image of license plate after plate conditioning.

The L-ANPR system consists of two cameras. The first camera is a 12.3 MP Raspberry Pi HQ model with a Sony IMX-477R sensor. The second camera is a 1.6 MP Raspberry Pi Global Shutter Camera with a Sony IMX 296 sensor. The L-ANPR detection system captures images with a resolution of 640 × 480 pixels. The low resolution is chosen to increase the frame rate per second to 90–120 to capture fast-moving L-vehicles. The detection system detects the license plates in the images and then crops the image to the size of the license plate. As moped plates are very small, the resolution of moped license plates is very low, and some characters are so blurred that it is difficult for the L-ANPR system to recognize them. Moreover, some characters merge with neighboring characters due to blurriness and are difficult to recognize, as shown in Figure 23. The blurriness and mixing of characters due to low resolution can decrease recognition efficiency by 30 to 40% for small moped license plates, depending on the camera’s placement relative to the vehicles’ passing position on the road. The solution to issues of low-resolution can is to use a camera that can take images at a high frame rate and with high resolution. Another solution is to develop an artificial intelligence-based character recognition and separation algorithm, which can be trained based on the field data collected during our validation and emission measurement campaigns.

#### 3.4.2. Effects of Shadow and Sunlight

**Figure 24:**
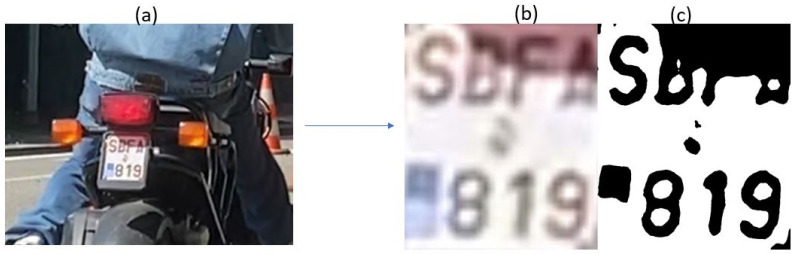
False recognition due to poor lighting and shadow: (**a**) Image of passing vehicle; (**b**) image of license plate after detection and cropping to region of interest; (**c**) image of license plate after plate conditioning.

The license plates of mopeds in some countries are tiny, and the characters even smaller. The visibility of such small characters is crucial for ANPR systems to recognize them correctly. Poor lighting conditions or strong sunlight can cast shadows or too much light on the license plates, which makes the visibility of tiny characters even more inadequate. Detecting tiny characters obscured by shadows is very difficult, as shown in Figure 24. When shadows cover the entire license plate but are of limited intensity, pre-processing techniques designed to mitigate the effects of adverse weather conditions can effectively address lighting and shadow-related issues. However, if shadows only affect certain characters with high intensity, it can complicate character recognition. The effects of shadows and sunlight can reduce recognition accuracy by 20% to 30%. One potential solution is to use cameras without an infrared (IR) cut filter, as well as to illuminate the entire license plates using IR light. This approach can also facilitate the detection and recognition of license plates at night.

#### 3.4.3. YOLOv4-Based Detection Algorithm

The L-ANPR detection algorithm is based on a CNN-based YOLOv4 real-time object detection model. Although the YOLOv4 is old, it is still considered one of the best options for real-time detection. The model was selected because it provides high accuracy, less computation cost, and is more efficient for single GPU training.

In 2025, YOLOv4 achieved the best performance relative to the state-of-the-art COCO dataset by combining advanced techniques for regression and classification using the Darknet framework. In the future, modified YOLOv4 or state-of-the-art CNN-based object detection algorithms, such as YOLOv7 and YOLOv8, and transformer-based models, like DETR and RT-DETR, can be utilized to develop license plate detection algorithms with even higher accuracy.

### 3.5. Adaptation of the L-ANPR System for Broader Applicability

**Figure 25:**
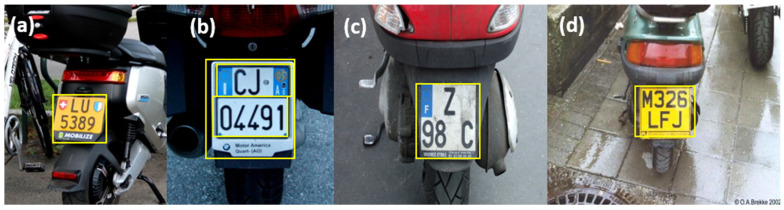
Application of L-ANPR license plate detection algorithm on license plates of L-vehicles of different countries: (**a**) Switzerland, (**b**) Italy, (**c**) France, and (**d**) United Kingdom.

The L-ANPR system has been trained using images of license plates from L-vehicles across various countries worldwide. To assess its adaptability, we used and tested the L-ANPR system with the license plates of several countries. The results, presented in Figure 25, demonstrate that the license plates of mopeds from France, Italy, Switzerland, and the United Kingdom were detected successfully. It can also be seen that the plates have favorable character-to-plate size ratios and are generally larger than the license plates of mopeds in Belgium and Austria. The images of license plates were collected from an online database [49].

Our findings indicate that Belgian mopeds feature some of the smallest plates, while Austrian mopeds have distinctly colored plates, displaying thin white characters on a red background, which differs from standard license plates globally, as illustrated in Figure 15 and Figure 16.

## 4. Summary and Conclusions

We present a cost-effective L-ANPR system, designed to detect and recognize the license plates of different types of L-vehicles in various categories. The L-ANPR system consists of three parts: L-ANPR light barriers, L-ANPR license plate detection system, and L-ANPR character recognition system. ANPR systems are essential for RES to identify high-emitting vehicles by retrieving key data from registration databases. Errors in ANPR can lead to invalid emission measurements. Recognizing number plates on L-vehicles (motorcycles, mopeds, quads, minicars) is especially challenging, as size, placement, and color may vary. The plates of some L-vehicles, i.e., mopeds, are often particularly small and difficult to read. While many ANPR systems exist, none are specifically designed for L-vehicles, and data on their performance with tiny, moped plates are limited.

The L-ANPR system utilizes light barriers, which consist of light transmitters and receivers, to identify Category L vehicles by measuring their size as they pass by. The L-vehicles are smaller than cars and trucks, allowing them to be distinguished based on size. Once the L-vehicles are recognized, the light barriers activate the L-ANPR detection system and other emission measurement devices, which then start monitoring. This technique significantly reduces energy consumption, data usage, and computational costs.

The L-ANPR license plate detection system is based on convolutional neural networks (CNN). It has been trained on thousands of images of license plates of different types of Category L-vehicles. The system is then tested on hundreds of images collected from online datasets, a validation campaign performed in Graz, Austria, and an emission measurement campaign performed at Leuven, Belgium. The overall detection accuracy for L-vehicle license plates was around 90%.

The L-ANPR recognition system consists of two parts: Conditioning and Extraction. Plate conditioning involves several steps: cropping, alignment, filtering, and thresholding. The extraction process also involves several steps: contour detection, masking, dilating, and deep learning-based optical character recognition. The system has been tested with hundreds of images of license plates of different L-vehicle types received from the L-ANPR license plate detection system. The images of license plates were obtained from online datasets and captured during a validation campaign conducted in Graz, Austria, as well as an emission measurement campaign in Leuven, Belgium. The overall recognition accuracy of license plates of L-vehicles was around 70%. The recognition rate is lower due to false and invalid recognitions caused by the lower resolution of pictures and the effects of shadow and sunlight during validation and emission measurement campaigns. Poor lighting conditions create shadows, and direct sunlight creates too much reflection on the license plates, which makes the visibility of tiny characters even more inadequate. Detecting tiny characters through shadows and reflection is very difficult. One possible solution is to use cameras without an IR-cut filter alongside the illumination of license plates with IR light. Low resolution is chosen because the cameras can capture a high frame rate per second only at lower resolution. This problem can be solved by using cameras that capture images at a high frame rate and with high resolutions.

In conclusion, there is no ANPR system specially designed for different types of Category L vehicles, and there is very little information on the usage of the ANPR systems on the tiny license plates of mopeds, the associated problems, and solutions. Generic state-of-the-art license plate detection and recognition algorithms were tested with different types of vehicles, and a performance comparison is presented. The algorithms performed well with cars but very poorly with L-vehicles. In this work, a cost-effective and energy efficient L-ANPR system is designed to detect and recognize the license plates of category L vehicles. The L-ANPR system first identifies category L vehicles by measuring their size as they pass. Once the L-vehicles are recognized, the light barriers activate the L-ANPR detection system, which then begins detecting license plates and recognizing license plate numbers. The L-ANPR system’s detection model is trained using thousands of images of license plates from various types of Category L vehicles across different countries. The L-ANPR system’s character recognition is designed to identify large characters on standard number plates and smaller characters in various colors on tiny, moped plates from different countries. The system’s performance is evaluated with an online dataset of license plates and the images captured during validation and emission measurement campaigns. In the future, the performance of the L-ANPR detection will be improved through more training with images collected from the emission measurement campaigns. Moreover, the performance of the L-ANPR recognition system will be improved by using high-speed and high-resolution cameras without IR-cut filters. Another approach is to create an AI-based character recognition and separation algorithm, trained using field data collected during our validation and emission measurement campaigns.

## Figures and Tables

**Table 1:** Configuration for L-ANPR detection model training.

Configuration Parameter	Value
Batch size	64
Subdivision	16
Epochs	100
Classes	1

**Table 2:** Performance metrics for L-ANPR detection model.

Performance Metric	Value
Precision	0.99
Recall	1.00
F1-score	0.99

**Table 3:** Overall detection accuracy of L-ANPR detection system.

L-Vehicle Type	Data Source	Detection Accuracy
Big Bikes or Scooters	Public dataset	~95%
Big Bikes or Scooters	Validation Campaign	~90%
Big Bikes or Scooters	Measurement Campaign	~90%
Mopeds	Public dataset	~90%
Mopeds	Validation Campaign	~85%
Mopeds	Measurement Campaign	~85%

**Table 4:** Overall recognition accuracy of L-ANPR character recognition system.

L-Vehicle Type	Data Source	Recognition Accuracy
Big Bikes or Scooters	Public dataset	~75%
Big Bikes or Scooters	Validation Campaign	~70%
Big Bikes or Scooters	Measurement Campaign	~70%
Mopeds	Public dataset	~70%
Mopeds	Validation Campaign	~65%
Mopeds	Measurement Campaign	~60%

## Data Availability

The data presented in this study are available on request from the corresponding author. The data are not publicly available due to privacy.
